# Metastatic Malignant Ovarian Steroid Cell Tumor: A Case Report and Review of the Literature

**DOI:** 10.1155/2016/6184573

**Published:** 2016-06-08

**Authors:** Jessica Lee, Veena S. John, Sharon X. Liang, Catherine A. D'Agostino, Andrew W. Menzin

**Affiliations:** ^1^New York University School of Medicine, Department of Obstetrics and Gynecology, Division of Gynecologic Oncology, 240 East 38th Street, New York, NY 10016, USA; ^2^Northwell School of Medicine, Hofstra University, Department of Hematology and Oncology, Lake Success, NY 11042, USA; ^3^Northwell School of Medicine, Hofstra University, Department of Pathology, Lake Success, NY 11042, USA; ^4^Northwell School of Medicine, Hofstra University, Department of Radiology, Manhasset, NY 11030, USA; ^5^Northwell School of Medicine, Hofstra University, Department of Obstetrics and Gynecology, Division of Gynecologic Oncology, Manhasset, NY 11030, USA

## Abstract

We report a case of malignant ovarian steroid cell tumor not otherwise specified (NOS) in a 47-year-old female who presented with hirsutism, virilization, and amenorrhea. At the time of laparotomy, the tumor had already spread to the pelvic cul-de-sac. She underwent a total hysterectomy, bilateral salpingo-oophorectomy, and tumor resection with no residual disease. She received three cycles of bleomycin, etoposide, and cisplatin (BEP) and is now free of disease 24 months after surgery. Literature review of ovarian steroid cell tumors NOS including clinicopathological features and clinical management was performed.

## 1. Introduction

Ovarian steroid cell tumors, also referred to as lipid or lipoid cell tumors, are rare and account for less than 0.1% of all ovarian tumors. These tumors are subdivided into stromal luteoma, Leydig cell tumor, and steroid cell tumor not otherwise specified (NOS). Steroid cell tumors NOS, tumors of uncertain cell lineage, comprise more than one-half of all ovarian steroid cell tumors. While almost all well-documented cases of stromal luteoma and Leydig cell tumor are clinically benign, up to 43% of steroid cell tumors NOS are clinically malignant [1]. Yet due to its rarity, only a limited number of cases have been reported in the English literature. Here we report a case of metastatic malignant steroid cell tumor NOS in a perimenopausal woman who presented with pelvic pain, hirsutism, and amenorrhea.

## 2. Case Report

A 47-year-old female, gravida 3, para 1, presented with pelvic pain. Patient reported significant hirsutism and amenorrhea for 5 years since the vaginal delivery of her daughter. The patient had been previously evaluated by an endocrinologist and diagnosed with noncongenital adrenal hyperplasia and polycystic ovarian syndrome. Baseline laboratory tests revealed an elevated testosterone level of 341.6 ng/dL (normal range 9.4–48.1 ng/dL) and androstenedione of 1,630 ng/dL (normal range 30–200 ng/dL), a normal dehydroepiandrosterone sulfate (DHEA-S) of 21.4*μ*g/dL (normal range 18–244*μ*g/dL), luteinizing hormone 1.4 IU/L (normal range 1.0–21.5 IU/L), estradiol of 56 pg/mL (normal range ≤ 388 pg/mL), and a low follicle-stimulating hormone 0.3 IU/L (normal range 15–290 IU/L) [Table 1]. She had been taking metformin for prediabetes and dexamethasone for the presumed adrenal hyperplasia without clinical improvement.

**Figure 1:**
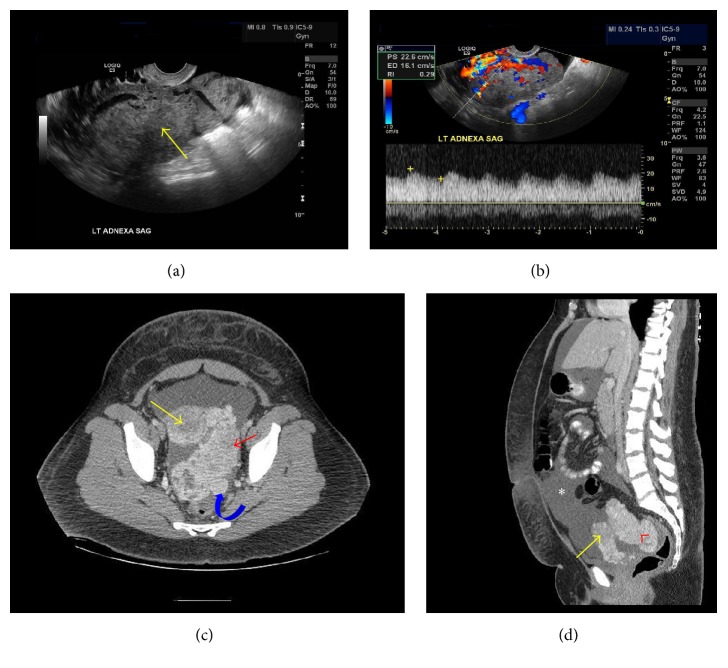
(a) Sagittal transvaginal ultrasound image: large heterogeneous adnexal mass. (b) Sagittal pelvic US with Doppler imaging: vascular adnexal mass with low resistant arterial flow (RI = 0.29). (c) Axial CT pelvis with IV contrast: yellow arrow: uterus, red arrowhead: enhancing adnexal mass, and curved blue arrow: cul-de-sac metastasis. (d) Sagittal CT pelvis with IV contrast: yellow arrow: uterus, red arrowhead: adnexal mass, and white asterisk: ascites.

Physical examination revealed an obese female with significant hair growth on her chest, back, four extremities, and face with male-pattern alopecia. Pelvic exam was significant for a 16-week sized mass posterior to the uterus. Pelvic sonogram revealed a solid left adnexal mass measuring 11.8 × 6.5 × 8.8 cm with marked increased vascularity and low resistance arterial waveforms with a resistance index of 0.29 (Figures 1(a) and 1(b)). A CT scan confirmed a 10.4 × 5.9 cm solid left adnexal mass as well as another solid nodule mass measuring 4.9 × 4 cm in the cul-de-sac and mild ascites (Figures 1(c) and 1(d)). Laboratory studies that had been sent by the referring gynecologist revealed a mildly elevated CA-125 level of 42 U/mL (normal range ≤ 34 U/mL), normal CA 19-9 of 4.7 U/mL (normal range ≤ 41.3 U/mL), and CEA of 1.9 ng/mL (normal range 0.0–3.8 ng/mL) [Table 1].

The patient underwent an exploratory laparotomy, and an 11 cm solid, friable left adnexal mass adherent to the pelvic sidewall and cul-de-sac was discovered along with copious amounts of yellow ascites. Total abdominal hysterectomy, bilateral salpingo-oophorectomy, omentectomy, and resection of cul-de-sac tumor with no gross residual disease were performed. Intraoperative frozen section of the left ovarian mass favored granulosa cell tumor.

Following final pathology diagnosis, the patient underwent three cycles of bleomycin, etoposide, and cisplatin (BEP). The patient received etoposide 100 mg/m^2^ and cisplatin 50 mg/m^2^ on days 1–5 and bleomycin 30 units on days 1, 8, and 15. She also received growth factor support with each cycle. Following chemotherapy, testosterone levels dropped to 3 ng/dL and CA-125 levels normalized at 5 U/mL. The patient reports significant improvement of her hirsutism. She is alive and free of disease 24 months after surgery.

## 3. Pathology

On gross examination, the left adnexa consisted of a normal fallopian tube attached to an 11 × 8 × 4 cm solid ovarian mass with a smooth intact surface. The sectioned surface was yellow to orange and showed a solid and multinodular appearance with focal hemorrhagic areas. Definite necrosis or cystic degeneration was absent. The uterus, cervix, right adnexa, and omentum were grossly normal. The fragmented cul-de-sac mass had an aggregate size of 2.0 × 1.5 × 0.5 cm comprised of friable, necrotic, dark hemorrhagic tissue.

**Figure 2:**
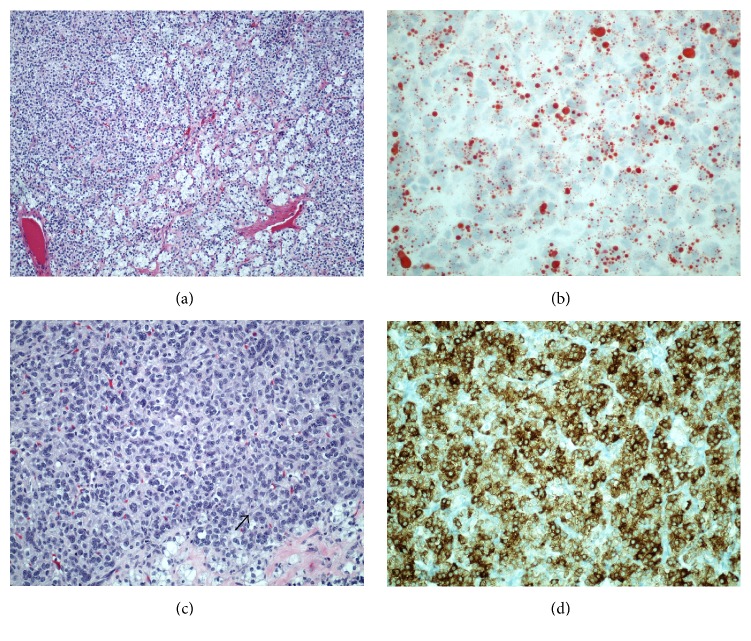
(a) The tumor demonstrated a solid and multinodular gross pattern composed of polygonal to round tumor cells (H&E stain). (b) The cytoplasm was moderate to abundant and varied from eosinophilic and granular (lipid-poor) to spongy which was lipid-rich as demonstrated by positive oil-red stain. (c) Arrow shows a representative cell with high tumor cellularity and a central nucleus containing prominent nucleoli. (d) Immunohistochemistry showed diffuse positive staining for inhibin.

On microscopic examination, the left ovarian mass demonstrated a solid and multinodular gross pattern composed of polygonal to round tumor cells with distinct cell borders (Figure 2(a)). The cytoplasm was moderate to abundant and varied from eosinophilic and granular (lipid-poor) to spongy which was lipid-rich as demonstrated by positive oil-red stain (Figure 2(b)). The nuclei were central and contained prominent nucleoli. There was grade 1 to 2 nuclear atypia (Figure 2(c)). Mitotic rate was up to 5 per 10 high-power fields. Hemorrhage was focally seen, but tumor necrosis was not identified. Immunohistochemistry showed diffuse positive staining for inhibin (Figure 2(d)) and negative staining for CD10, PAX-8, and CK7. The cul-de-sac tumor showed similar tumor morphology to extensive hemorrhage and necrosis.

## 4. Discussion

Ovarian steroid cell tumors are rare and account for less than 0.1% of all ovarian tumors. These tumors are subdivided into stromal luteoma, Leydig cell tumor, and steroid cell tumor NOS. The former two categories each account for 20% of steroid cell tumors and arise from either ovarian stroma or hilar cells. Steroid cell tumors NOS are tumors of uncertain lineage and comprise approximately 60% of steroid cell tumors. Many of these tumors have been described in the literature as case reports (Table 2). Hayes and Scully reported the only largest case series in 1987 of 63 cases [1].

Steroid cell tumors NOS typically present in adults with an average age at diagnosis of 47 years. The majority of patients (56–77%) present with hirsutism and virilization, secondary to the production of testosterone from the tumor. These tumors can also secrete estradiol, which has been reported in 6–23% of patients in Hayes and Scully's case series [1]. This excess of estrogen can result in menorrhagia or postmenopausal bleeding and ultimately endometrial adenocarcinoma [2]. There are reports of cortisol release by these tumors, and these can present in a clinical picture similar to Cushing's syndrome [3, 4]. Approximately 25% of steroid cell tumors NOS do not produce any hormones.

Serum laboratory analysis typically reveals elevated testosterone and androstenedione levels that indicate an ovarian origin of androgen release and normal DHEA-S levels that rule out an adrenal etiology for the hyperandrogenism [5]. Tumor markers such as CA-125, CEA, and CA 19-9 are typically not helpful in the workup. The utility of newer diagnostic factors including HE4 and Risk of Ovarian Malignancy Algorithm (ROMA) has not yet been established in the preoperative workup for steroid cell tumors. Imaging including CT of the adrenals and ovaries can identify an ovarian or adrenal mass. There have been reports of small aberrant masses in the parametria that can go missed on imaging. MRI has been shown to have diagnostic value with the tumor's lipid components showing intense enhancement on chemical shift MRI [6, 7].

Steroid cell tumors NOS are clinically malignant in 25–43% of the tumors. In Hayes and Scully's series, 24 of the 42 cases were probably benign based on no evidence of extra-ovarian spread within 3 or more years after surgery, while tumors in the remaining 18 cases were clinically malignant (43%). The best pathological correlates of malignant behavior were ≥2 mitotic figures per 10 high-power fields (92%), necrosis (86%), tumor size ≥ 7 cm (78%), hemorrhage (77%), and grade 2-3 nuclear atypia (64%) [1]. The tumor in our case demonstrated all these pathologic features except necrosis.

Steroid cell tumors NOS are primarily managed surgically as with other ovarian stromal tumors. For early stage tumors, conservative surgery with unilateral oophorectomy and proper staging is an option in patients who desire future fertility. For those who have completed childbearing, total hysterectomy, bilateral salpingo-oophorectomy, and complete staging are indicated. For malignant tumors with advanced stage, adjuvant therapy is suggested.

There is no consensus on the adjuvant therapy for metastatic malignant steroid cell tumors NOS. The Gynecologic Oncology Group has shown that BEP is effective as the first line treatment for malignant ovarian stromal tumors. In patients with stage II primary ovarian stromal tumors or recurrent disease, 69% were free of disease after four cycles of BEP [8]. There have been promising reports with the use of GnRH-agonists as primary adjuvant therapy for sex cord stromal tumors including steroid cell tumors [9, 10]. Testosterone levels can be monitored for disease progression or recurrence.

In summary, steroid cell tumors NOS are rare ovarian neoplasms but should be considered in patients who present with hirsutism and elevated testosterone levels. Surgical removal is the mainstay of treatment. The pathological characteristics identified by Hayes and Scully differentiate benign and malignant tumors. Adjuvant therapy with BEP chemotherapy or GnRH-agonist therapy is indicated for metastatic malignant steroid cell tumors NOS.

## Figures and Tables

**Table 1:** Values of hormone/tumor marker levels.

Marker/hormone	Test value	Normal values
Testosterone	341.6 ng/dL	9.4–48.1 ng/dL
Androstenedione	1630 ng/dL	30–200 ng/dL
Dehydroepiandrosterone sulfate (DHEA-S)	21.4 *μ*g/dL	18–244 *μ*g/dL in 40–49 years
17-Hydroxyprogesterone	588 ng/dL	15–290 ng/dL
CA-125	42 U/mL	≤34 U/mL
CA 19-9	4.7 U/mL	≤41.3 U/mL
Carcinoembryonic antigen (CEA)	1.9 ng/mL	0.0–3.8 ng/mL

**Table 2:** Documented cases of malignant steroid cell tumor NOS with references, ordered by year of publication.

Reference	Age at diagnosis	Presentation	Initial surgery	Stage at diagnosis	Postoperative treatment	Recurrence	Survival (months)
Hayes and Scully (18 cases) [1]	2.5 to 80 (average 43)	Hirsutism, virilization, postmenopausal bleeding, menorrhagia, abdominal swelling	TAH, BSO, or USO	I–IV	Vincristine, actinomycin D, mitotane, cisplatin, doxorubicin, cyclophosphamide, 5-fluorouracil, radiation	Yes in 4 of 18 cases	5–228

Donovan et al. [3]	66	Abdominal enlargement, leg edema, hypertension	TAH, BSO, omentectomy, appendectomy, PPALND, debulking	IV	Cisplatin, methotrexate, vinblastine then enalapril, ketoconazole, etoposide, ifosfamide	Yes	Not reported but died of disease

Wang et al. [9]	50	Hirsutism	TAH, BSO, omentectomy, PPALND	IIA	GnRH-agonist	None	32

Wang et al. [6]	48	Hirsutism	TAH, BSO, omentectomy, PPALND	IIA	None	None	6

Brewer and Shevlin [10]	58	Pelvic pain, virilization	TAH, BSO, PPALND	IIIC	BEP with progression after second cycle then GnRH-agonist	Yes	11

Garduño-López et al. [11]	34	Abdominal pain, hirsutism	Hepatic trisegmentectomy	IV	BEP	None	6

Saida et al. [7]	28	Virilization	USO	IA	None	Unknown	Unknown

Murhekar et al. [12]	31	Hirsutism, oligomenorrhea	Resection of isolated mass on pelvic mesentery	IA	None	Unknown	Unknown

Jiang et al. [13]	21	Amenorrhea, virilization	TAH, BSO, omentectomy, debulking of peritoneal metastases	IIIC	BEP	Yes	10

Li et al. [14]	29	Back and leg pain	USO	IV	Docetaxel and nedaplatin	Yes	6

TAH: total abdominal hysterectomy.

BSO: bilateral salpingo-oophorectomy.

PPALND: pelvic and para-aortic lymph node dissection.

USO: unilateral salpingo-oophorectomy.

GnRH: gonadotropin-releasing hormone.

BEP: bleomycin, etoposide, and cisplatin.
